# Co-existing Gastrointestinal Hemorrhage and Deep Vein Thrombosis in a Patient with Hereditary Hemorrhagic Telangiectasia: Management Dilemma

**DOI:** 10.7759/cureus.3305

**Published:** 2018-09-14

**Authors:** Hasnan M Ijaz, Muhammad Uzair Lodhi, Waliul Chowdhury, Intekhab Askari Syed, Chirag Patel, Bryce A. McDaniel, Mustafa Rahim

**Affiliations:** 1 Internal Medicine, Raleigh General Hospital, Beckley, USA; 2 Preventive Medicine, Raleigh General Hospital, Beckley, USA; 3 Family Medicine, Beckley Appalachian Regional Healthcare, Beckley, USA; 4 Internal Medicine, Charleston Area Medical Center / West Virginia University, Charleston, USA; 5 Internal Medicine, West Virginia University School of Medicine, Morgantown, USA

**Keywords:** hereditary hemorrhagic telangiectasia, osler-weber-rendu disease, gastrointestinal hemorrhage, deep vein thrombosis

## Abstract

Hereditary hemorrhagic telangiectasia (HHT) is described as a vascular defect, causing recurrent visceral and mucocutaneous bleeding. It is an autosomal dominant disease and has variable expressivity. The phenotypic presentation is dependent on the type of gene defect. Recurrent epistaxis is the most common symptom, along with gastrointestinal (GI), pulmonary, and arteriovenous malformations (AVM). The Curacao criteria are used to make the diagnosis of HHT. Genetic sequence testing for endoglin (ENG) or activin receptor-like kinase type 1 (ALK1) can be performed to confirm the diagnosis. However, genetic sequencing is not necessary. Along with recurrent bleeding, patients with HHT also have an increased risk of thromboembolic events. Supportive treatment prevents acute symptoms, but the therapeutic options of HHT are based on multiple factors. We describe the case of a 69-year-old male who presented with GI bleeding and a history of HHT and recurrent deep vein thrombosis (DVT). We discuss the diagnostic guidelines and treatment options for patients with HHT. Furthermore, we also discuss the challenge in treating patients with co-existing GI bleeding and DVT, as in our case.

## Introduction

The incidence of an autosomal dominant disorder known as hereditary hemorrhagic telangiectasia (HHT) or Osler-Weber-Rendu disease is one in 8000 people. The complex pathophysiology of HHT includes molecular abnormalities that disrupt the cellular pathways, leading to abnormal vascular development. Patients with HHT have various defective genes, but the three most common are endoglin (HHT1), activin receptor-like kinase (ALK1/HHT2), and SMAD4 (HHT associated with juvenile polyposis) [1]. Endoglin and ALK1 upregulate transforming growth factor beta (TGF-B) on endothelial cells, which promotes angiogenesis and helps vascular homeostasis [2]. A mutation of endoglin or ALK1 makes vessels vulnerable to damage. Atypical vessel formation causes recurrent epistaxis and GI bleeding, which leads to anemia. Dilated mucocutaneous blood vessels are often evident on physical examination. A review of the patient's history, physical examination, and laboratory workup is necessary. The diagnosis of HHT is made by using the Curacao criteria [3].

## Case presentation

A 69-year-old Caucasian male presented with a complaint of constant fatigue and weakness for multiple years. The patient had a history of epistaxis since childhood. According to the patient, tilting his head backward exacerbated the nosebleed and sitting upright alleviated the bleeding. The patient also had multiple first- and second-degree relatives with arteriovenous malformations and epistaxis. The patient also complained of a productive cough with clear sputum for the past six months. Additionally, the patient reported having exertional dyspnea and intermittent paroxysmal nocturnal dyspnea. His past medical history consisted of anemia, GI bleeding, gastric ulcer, melena, diabetes mellitus type 2, bilateral DVT, hypertension, arteriovenous malformation of the small bowel, occasional orthostatic lightheadedness, and scarlet fever. Past surgical history included multiple esophagogastroduodenoscopy (EGD) procedures.

**Figure 1:**
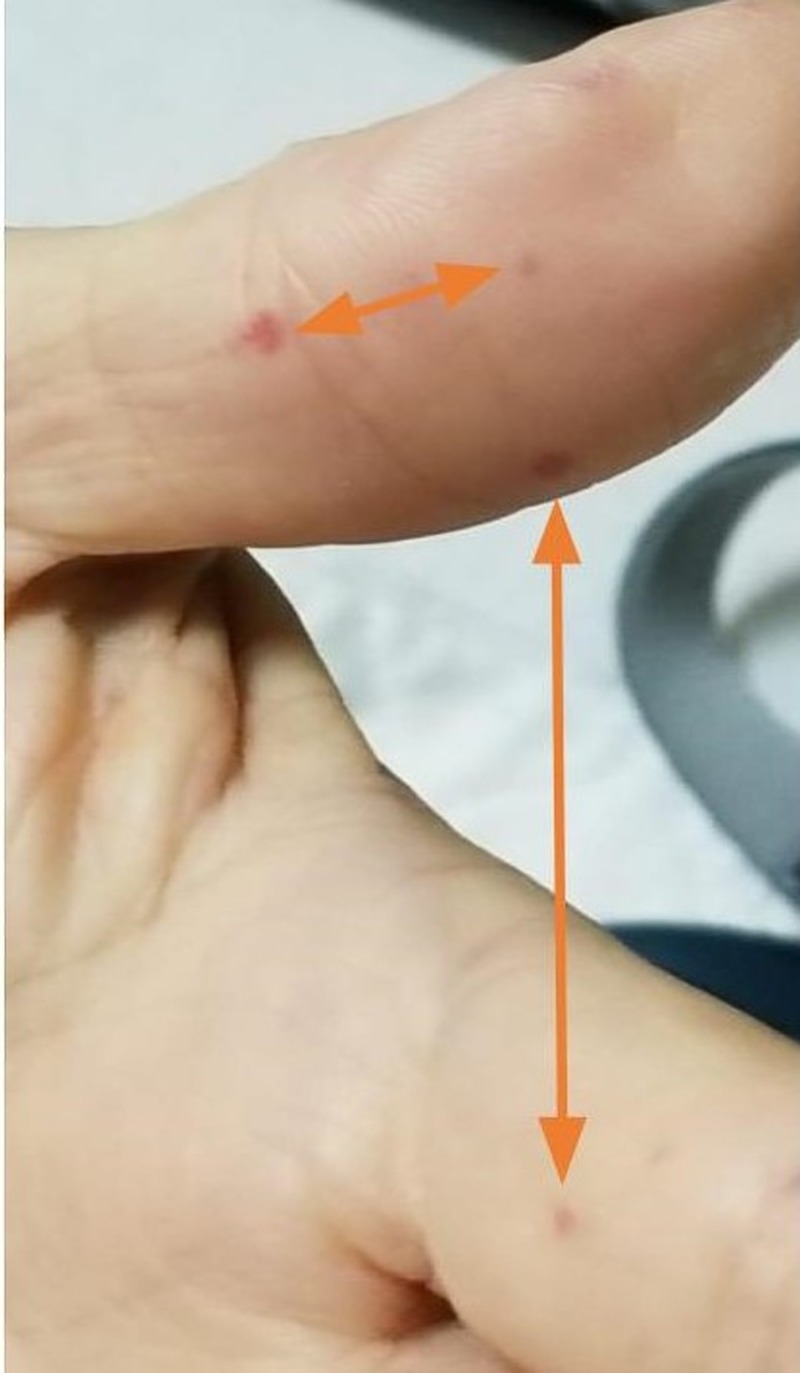
Telangiectasias of the fingers (orange arrows)

**Figure 2:**
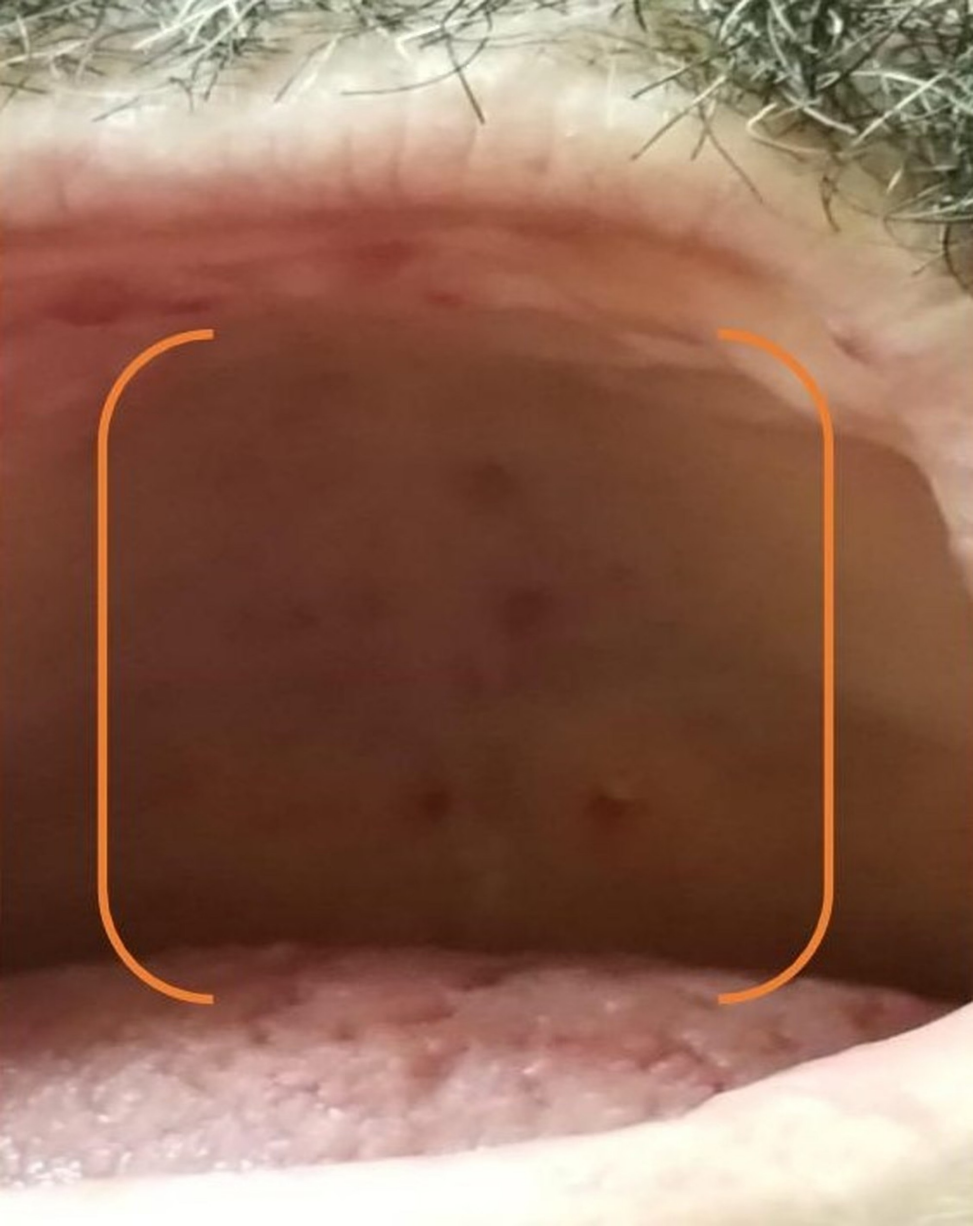
Telangiectasias of the palate (orange bracket)

**Figure 3:**
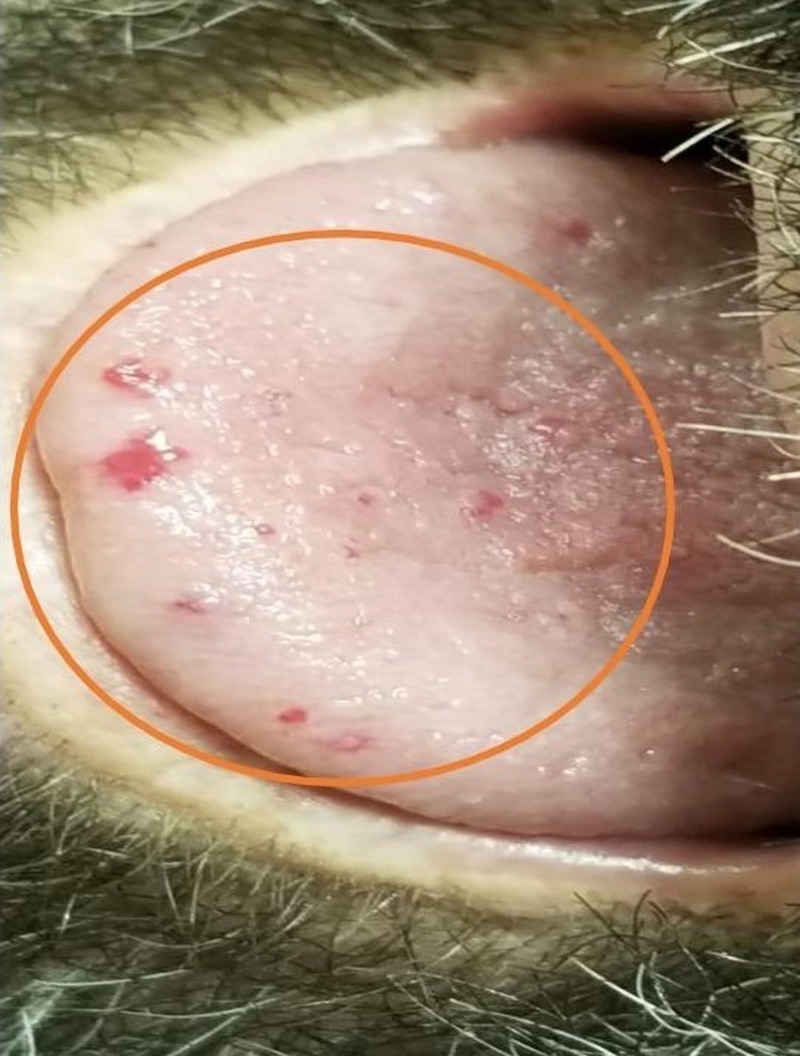
Telangiectasias of the tongue (orange circle)

**Figure 4:**
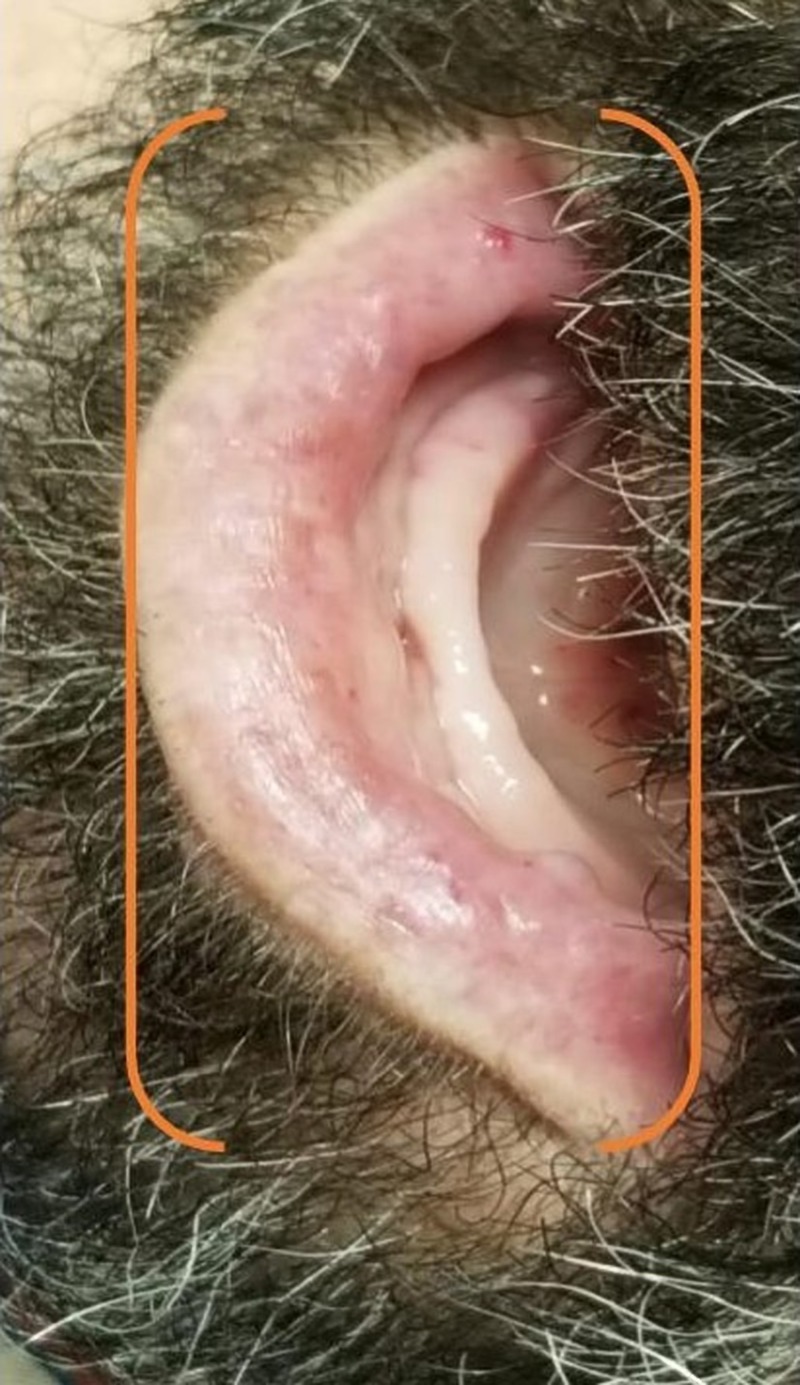
Telangiectasias of the lower lip (orange bracket)

**Figure 5:**
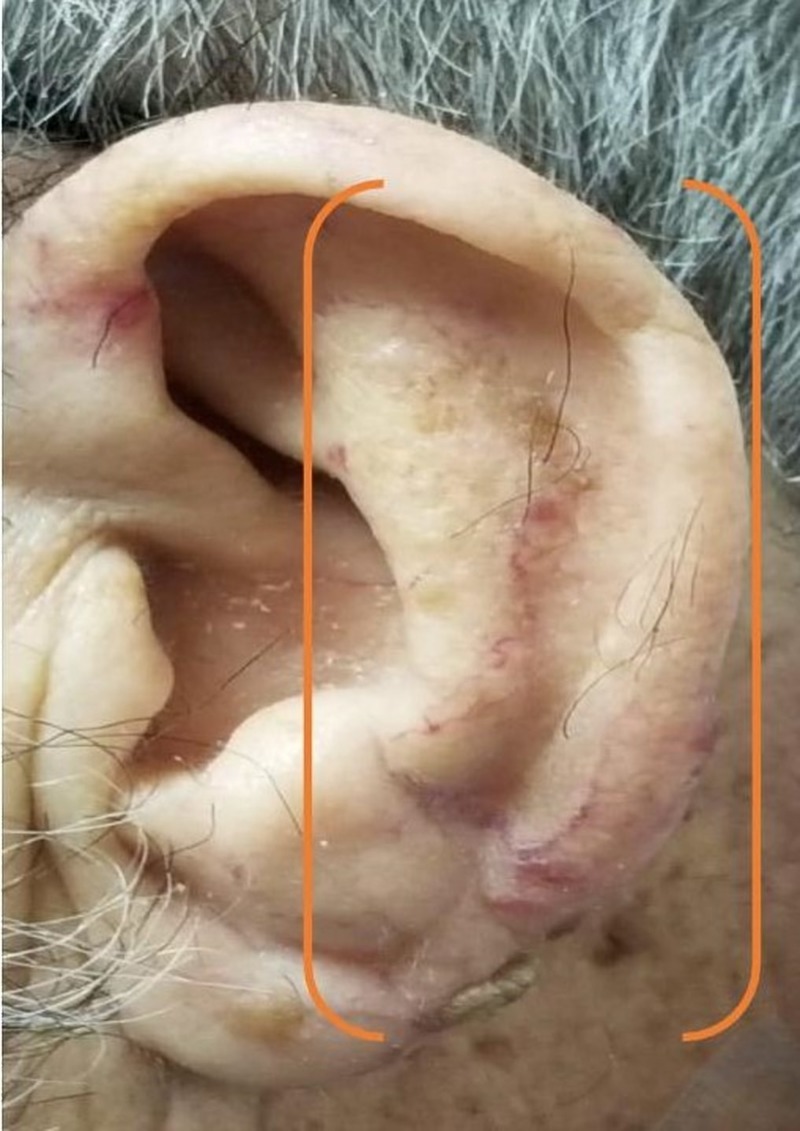
Telangiectasias of the ear (orange bracket)

**Figure 6:**
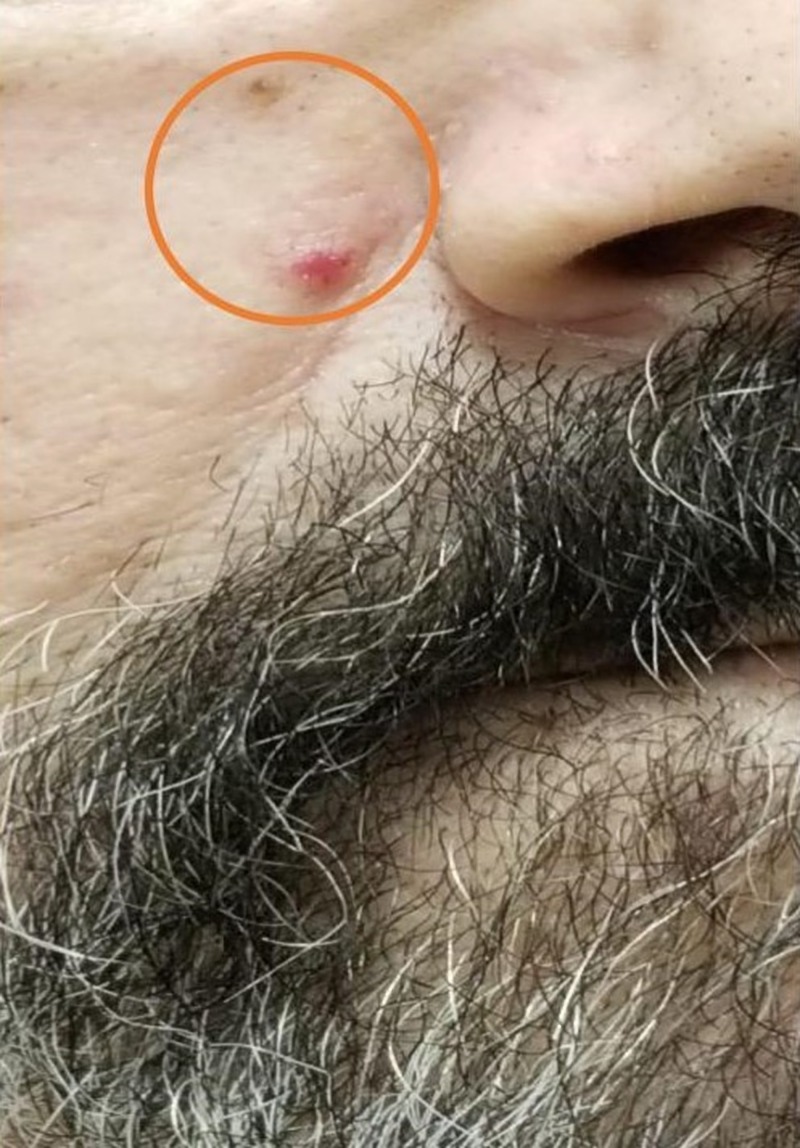
Telangiectasias of the face (orange circle)

Upon physical examination, the patient was not in acute distress. His vitals were as follows: a blood pressure of 119/70 mmHg, a pulse of 68 beats per minute (bpm), a temperature of 103 F, and a respiratory rate of 16 breaths per minute (bpm). At presentation, the patient had multiple vascular malformations on the fingers, upper palate, tongue, lower lips, ears, and the face (as shown in Figures 1-6, respectively). The S1 and S2 sounds were audible with a regular rate and rhythm. The tenderness was present on deep palpation in the left lower quadrant. There was no leg swelling, warmth, or redness. Peripheral pulses were palpable. The deep tendon reflex was normal and the cranial nerves were intact.

Hospital course

Considering the consistently low hemoglobin (Hb), fatigue, and occult positive stool results, the patient was admitted to the hospital and was given two units of blood. The epistaxis episode resolved spontaneously. On day one, the patient's Hb level was 4.9, and he was treated with four units of packed red blood cells (PRBC), and the Hb increased to 8.0. On day two, the patient's Hb dropped to 7.7, and he was infused with another unit of PRBC. The colonoscopy result showed bleeding in the ascending colon. The patient also underwent esophagogastroduodenoscopy (EGD), showing multiple 4-millimeter angiodysplasias with active bleeding at the cardia and fundus. There were also non-bleeding angiodysplasias in the duodenum and the jejunum, which were treated with argon beam coagulation and photodynamic therapy.

## Discussion

The Curacao criteria are established by the scientific advisory board of the HHT Foundation International. The criteria are characterized by recurrent epistaxis, visceral bleed, mucocutaneous telangiectasia, and a family history of HHT. The presentation of three or more symptoms is required to establish a definitive diagnosis. Patients presenting with two symptoms are considered at risk of developing the disease [2]. Epistaxis and GI bleeding are the most common causes of anemia in HHT. The patient is often born without symptoms. Epistaxis is more common in younger patients while GI bleeding presents in the fifth decade of life. Frequent rupture of angiodysplasias and blood loss worsens in adults. In one of the studies, gastrointestinal bleeding was reported in 33% of the patients with HHT. The same study also reported that 25% of HHT patients greater than 60 years of age suffered from severe gastrointestinal bleeding [4]. Additionally, patients with HHT not only have a higher bleeding tendency but they also have an increased risk of thromboembolism. A study conducted by Livesey et al. reported that the risk of thromboembolism increased by almost two-fold in a patient with HHT compared to the general population. Recurrent bleeding in HHT decreases serum iron levels. The low iron has an inverse relationship with factor VIII and contributes to these patients having a 2.5-fold increased risk of venous thromboembolism [5]. The management of HHT is a complicated and frustrating process for both patient and physician. Multiple bleeding events lead to iron deficiency anemia. The patient is often given a dietary plan, which includes an iron-rich diet and an iron supplement. Even with a proper diet, the patient often requires a regular blood transfusion. Therapeutic decisions are based on the bleeding symptoms, but the challenge arises in a patient with DVT. To our knowledge, in the current literature, there is no definitive treatment for GI bleeding in an HHT patient with a history of recurrent DVT.

Hormonal treatment has been used to decrease recurrent epistaxis episodes and GI bleeding. Tamoxifen or raloxifene work at a molecular level by upregulating multiple genes. Their effect is on the transcription factor at the promoter region of endoglin and ALK1 through an increased expression of their receptors on the cell surface. The upregulation of these proteins has a pro-angiogenic effect on vessels [6]. It may decrease the bleeding complication, but it is a contraindication in our patient due to its adverse effect of potentially exacerbating the DVT.

Thalidomide and bevacizumab are antiangiogenic drugs that have shown to improve recurrent nose bleeds and incidences of telangiectasia. Bevacizumab affects the vascular endothelial growth factor (VEGF) receptors and prevents abnormal angiogenesis. Long-term treatment with intravenous bevacizumab has shown an improvement in the cessation of bleeding and helps improve the hemoglobulin level. Similarly, thalidomide not only affects VEGF but also downregulates TGF-B. Along with angiogenesis, thalidomide also has an anti-inflammatory property that provides additional benefits in a patient with HHT. Thalidomide also decreases the epistaxis and improves the telangiectasia spots. Despite the improvement in bleeding problems, the severe adverse effect has been seen in patients who are taking bevacizumab and thalidomide. The study conducted by Cohen et al. described several adverse effects of bevacizumab, such as an increased risk of arterial thromboembolic events, delayed wound healing, hemorrhage, and gastrointestinal perforation. Thalidomide also causes thromboembolic complications [7-10].

Argon plasma coagulation (APC) is a medical endoscopic procedure used primarily to control bleeding from certain lesions in the gastrointestinal tract by a high-frequency current distributed on the tissue through ionizing argon gas. Kwan et al. conducted a prospective study, which showed an improvement in hemoglobin levels and a decrease in the recurrence of blood transfusions in a patient with APC treatment [11]. Immediate hemostasis rates range from 85 to 100 percent in various reports and treatment can result in the long-term control of bleeding [11-14].

In hospitals, a patient with current DVT and a history of GI bleeding (with HHT) is often treated with short-term anticoagulant or antiplatelet therapy. The extended use of anticoagulant therapy is a contraindication due to severe hemorrhagic complications.

## Conclusions

Chronic GI bleeding causes low iron, which increases the risk of DVT. Once the disease progresses, the treatment to control recurrent bleeding and DVT becomes more laborious. The use of currently available hormonal medications for HHT becomes a contraindication due to the risk of DVT. Regular monitoring for anemia and ultrasounds to assess for DVT formations in these patients are often required. Early preventative treatment for patients who have first-degree relatives with HHT can prevent delayed complications. We hope our case report highlights the management dilemma in treating co-existing GI bleeding and DVT in a patient with HHT. Furthermore, we also hope that it possibly helps dictate the further research needed for the early diagnosis and management of these patients.
